# Renal outcomes in the long-term follow-up of lupus nephritis

**DOI:** 10.1590/2175-8239-JBN-2024-0118en

**Published:** 2025-04-14

**Authors:** Sabrina Bonvino Polycarpo, Gianna Mastroianni Kirsztajn

**Affiliations:** 1 Universidade Federal de São Paulo, Departamento de Medicina (Nefrologia), São Paulo, SP, Brazil.

**Keywords:** Lupus Nephritis, Lupus Erythema-tosus, Systemic, Renal Insufficiency, Chronic, Kidney Failure, Chronic, Antimalarials, Creatinine, Glomerular Filtration Rate

## Abstract

**Introduction:**

Lupus nephritis (LN) is one of the most common and serious manifestations of systemic lupus erythe-matosus (SLE) and is recognized as the strongest predictor of poor prognosis. This study aims to analyze demographic characteristics, clinicopathological corre-lations, and risk factors associated with renal outcomes in a long-term follow-up of patients with LN.

**Method:**

This is a retrospective observational cohort study from 112 biopsy-proven LN patients, followed from July 1985 to August 2015. We have evaluated risk factors associated with renal impairment (doubling of serum creatinine), end-stage renal disease (ESRD, eGFR <15 mL/min) and cumulative renal damage using the Systemic Lupus International Collaborating Clinics/ACR Damage Index (SDI).

**Results:**

The median time of follow-up was 8.3 years. Class IV LN predominated (64.3%), alone or in combination. There was a statistically significant increase in the renal score of SDI between 5 and 10 years of follow-up. In the multivariate analysis, final SDI correlated with initial eGFR <75 mL/min (p < 0.001) and proteinuria >3.5 g/24 h during the course of the disease (p < 0.001), overcoming parameters identified in uni-variate analysis as using cyclophosphamide (p = 0.010) and not using antimalarials (p = 0.003); 13.4% of the patients progressed to renal function impairment, and 11.6% to ESRD.

**Discussion:**

Accumulated renal damage by SDI was a frequent finding. The main determinants of renal outcome were baseline eGFR <75 mL/min and proteinuria >3.5 g/24 h during follow-up. Thus, early diagnosis and better management of LN, especially in case of high levels of proteinuria, may help improve prognosis.

## Introduction

Systemic lupus erythematosus (SLE) is a chronic, multisystem autoimmune disease of the connective tissue^1^. Renal involvement in SLE, lupus nephritis (LN), is frequent and is also one of the most serious manifestations of SLE. It affects up to 60% of adults with the disease; the incidence depends on the population studied, with a higher incidence in Asians, Afro-descendants and Hispanics^2,3,4^. LN is recognized as the strongest predictor of a poor prognosis, since it is associated with a significant increase in mortality^2,3,4,5,6^. Approximately 50 years ago, the median five-year survival of patients with LN was 44%; it improved dramatically to approximately 82% by the 1990s. Such a decrease in mortality is due to better knowledge of the disease, leading to earlier diagnosis and better and more aggressive therapeutic management^2,4,7^.

Despite improving the course of the disease, treatment may be prolonged, potentially toxic, and difficult to plan and maintain^8,9^. Both treatment and disease activity over time can result in permanent damage to different organs and lead to dysfunction, such as chronic kidney disease (CKD) in the case of LN. For some authors^9,10^, the identification of predictors of cumulative damage is of great importance to improve SLE outcomes. In this context, the Systemic Lupus International Collaborating Clinics/ACR Damage Index (SDI) is a validated instrument to measure irreversible damage in SLE patients^9^. In fact, short-term survival of patients with SLE has improved, but irreversible organ damage accumulates during the course of the disease, contributing to impaired quality of life and increased long-term mortality. Thus, patient management should aim not only to increase survival but also to reduce morbidity caused by the disease and its therapy^10^. This can be in part evaluated by indexes like SDI, that can provide additional information in the follow-up of patients with LN.

The average incidence of end-stage renal disease (ESRD) in 10 years of follow-up is 15% and may reach 25−30% in 15 years^11,12,13,14,15^. Lack of remission of LN with treatment is probably one of the most important predictors of progression to CKD. Other risk factors for renal impairment have also been established and include male gender, low socioeconomic status, ethnicity (Afro-descendants and Hispanics), presence of crescents in more than 50% of the glomeruli in renal biopsy, proliferative classes (III and IV) and their treatment with corticoids alone, not using antimalarials, high initial serum creatinine, nephrotic proteinuria, hypertension, anemia, and complement consumption^16,17,18,19^. Such characteristics tend to vary according to each study, since there are differences in outcome definitions, populations, and duration of follow up, making it difficult to extrapolate findings.

Previous studies on outcomes in Brazilian patients with LN showed that initial proteinuria and serum creatinine levels, as well as initial response to treatment and chronicity on kidney biopsy were related to worse outcomes^20,21,22^. We analyzed demographic characteristics, clinicopathological correlations, and risk factors associated with renal outcomes in a long-term follow-up, including loss of renal function, ESRD, and cumulative damage in a longitudinal cohort of biopsy-proven LN patients.

## Methods

We analyzed retrospective data from medical records of patients with a diagnosis of SLE according to the revised criteria of the American College of Rheumatology (ACR) and histopathological diagnosis of LN, followed up at the Division of Nephrology (Section of Glomerulopathies) of the Federal University of São Paulo (UNIFESP), a tertiary and academic reference center in São Paulo, Brazil. The patients included were followed from July 1985 to August 2015. LN was classified according to the World Health Organization (WHO) and the International Society of Nephrology/Renal Pathology Society (ISN/RPS) classifications^23^. Patients of both genders, 12 years of age or older, and with a time of follow-up equal to or greater than one year were included.

The following variables were evaluated: age at the onset of follow-up, gender, blood pressure, serum creatinine, proteinuria, hematuria, antinuclear and anti-double stranded DNA (anti-dsDNA) antibodies, complement consumption, laboratory and clinical manifestations of SLE, estimated glomerular filtration rate (eGFR) determined using the Chronic Kidney Disease Epidemiology Collaboration (CKD-EPI) formula,^24^ and biopsy reports. The type and duration of treatment and the time of onset of infections were also recorded.

Renal impairment was defined as at least doubling serum creatinine level and ESRD as eGFR <15 mL/min (CKD stage 5) or when renal replacement therapy was required. We also assessed renal damage using the SDI^9^. This instrument has already been validated and applied in various populations and ethnicities and evaluates the nonreversible damage related to SLE and its treatment in 12 organs or systems^9,10,25^. Damage in the renal system according to SDI was defined as one of the following: 1) estimated GFR <50 mL/min (score: 1); 2) persistent nephrotic-range proteinuria ≥ 3.5 g/24 h (score: 1); and ESRD (score: 3). All patients had the SDI assessed at 1 year and at the end of follow-up; 34 patients who were followed for a longer period also had the SDI assessed at 5 and 10 years.

Statistical analyses were initially performed descriptively (means, medians, percentages), then inferential tests were applied when necessary and cited in the Results section. P-values lower than 0.05 were considered statistically significant. Analyses were carried out using R software version 3.0.2. (R Core Team, 2016).

## Results

A total of 112 patients were included, with a median follow-up time of 99.9 months (range 12−322). The mean age was 31 ± 10.8 years, 83.9% were females, and 54.8% were white. The baseline characteristics are shown in Table 1.

**Table 1:** Demographic and clinical characteristics of the patients with lupus nephritis (n = 112)

Mean age ± SD (years)	31 ± 10.8
Gender (%)	
Male	16.1
Female	83.9
Mean follow-up time (months)	99.9 (12-322)
Race (%)	
White	54.8
Non-white	45.2
Mean baseline serum creatinine ± SD (mg/dL)	1.62 ± 1.30
Mean eGFR ± SD (mL/min/1.73 m^2^)	71.1 ± 40.1
Final eGFR (%)	
≥ 15 mL/min	87.5
< 15 mL/min	12.5
Mean proteinuria ± SD (g/24 h)	4.15 ± 3.34
Proteinuria during follow-up (%)	
> 3.5 g/24 h	72.3
≤ 3.5 g/24 h	27.7
Hematuria at presentation (%)	87.5
Hematuria during follow-up (%)	
≤ 100,000/mL	34.8
> 100,000/mL	65.2
Dialysis for AKI during follow-up (%)	16.1
Hypertension (%)	76.8
Anemia (%)	74.1
Thrombocytopenia (%)	8.0
Leukopenia (%)	15.2
Complement consumption (%)	68.8
Anti-dsDNA (%)	15.2

The predominant clinical manifestations of SLE associated with LN were hematological (80.4%), especially anemia (74.1%), followed by complement consumption (68.8%), and joint involvement (67%). Hematuria >100,000/mm^3^ at presentation of renal disease occurred in 52 patients (53.1% of those with hematuria) and at the end of follow-up in six patients (11.1% of those with hematuria at this stage). At presentation, the laboratory profile showed elevated serum creatinine (mean of 1.62 mg/dL) and proteinuria >3.5 g/24 h in 49% of the patients and, at some point in the course of disease, in 72% of the patients.

The distribution of the histological classes of LN was as follows: 3.57% class II, 7.14% class III, 64.29% class IV, 23.21% class V, and 1.79% class VI. A combination of classes occurred in 11 patients, all with class IV, and 8 of them were class IV+V. For simplicity, the latter were all categorized as class IV.

Hypertension was diagnosed in 76.8% of the patients, requiring only one or two antihypertensive medications for control in most cases. All patients had received steroids during follow-up; 53.6% of the patients had received intravenous cyclophosphamide, 19.6% oral cyclophosphamide, 47.3% azathioprine, 41.1% mycophenolate (mofetil or sodium), and 62.5% antimalarials (chloroquine or hydroxychloroquine). Treatment with azathioprine was more common in patients with class II LN (p = 0.047). In class IV, the most commonly used treatments were intravenous cyclophosphamide (p < 0.001) and mycophenolate (p = 0.003), while these treatments were less used among patients with class V (p = 0.006 and p = 0.011, respectively). Infections of the upper respiratory tract were associated with the use of oral cyclophosphamide (p = 0.034).

Compared to the other LN classes, class IV LN (Table 2) was associated with worse renal function at baseline, higher initial serum creatinine (p < 0.001), and lower initial eGFR (p < 0.001), as well as more frequent detection of renal activity criteria, such as initial and follow-up hematuria >100,000/mL (p < 0.001), immunological activity with a higher frequency of anti-dsDNA positivity (p < 0.001), and more frequent detection of hypertension (p = 0.028). Class IV also presented more ESRD at the end of the follow-up when compared with the other classes (p = 0.003).

**Table 2:** Distribution of the demographic, clinical, and laboratory profile of the patients with class iv lupus nephritis

		LN class IV		
		yes	no	P
Gender	male	61.1%	38.9%	0.759^c^
	female	64.9%	35.1%	
	Total	64.3%	35.7%	
Age (years)	n	72	40	0.417^b^
	mean	30.3	32.3	
	median	28.0	30.5	
	minimum	13.0	14.0	
	maximum	54.0	73.0	
	standard deviation	10.3	11.6	
Race	white	71.9%	28.1%	0.078^c^
	non-white	55.3%	44.7%	
	Total	64.4%	35.6%	
Initial serum creatinine (mg/dL)	n	72	40	<0.001^b^
	mean	2.03	0.88	
	median	1.40	0.80	
	minimum	0.43	0.44	
	maximum	7.00	1.84	
	standard deviation	1.45	0.33	
Initial eGFR (mL/min)	n	72	40	<0.001^b^
	mean	56.34	97.77	
	median	50.55	99.25	
	minimum	7.30	42.70	
	maximum	145.40	151.00	
	standard deviation	37.12	30.54	
24-h proteinuria during follow-up	≤ 3.5g/24h	58.1%	41.9%	0.395^c^
	>3.5g/24h	66.7%	33.3%	
	Total	64.3%	35.7%	
Initial 24-h proteinuria	0.2 - 2g/24h	62.9%	37.1%	0.219^c^
	2 - 3.5g/24h	50.0%	50.0%	
	>3.5g/24h	70.9%	29.1%	
	Total	64.3%	35.7%	
Initial hematuria (if present)	10,000 - 100,000/mL	50.0%	50.0%	<0.001^c^
	>100,000/mL	84.6%	15.4%	
	Total	68.4%	31.6%	
Hematuria during follow-up	≤ 100,000/mL	41.0%	59.0%	<0.001^c^
	>100,000/mL	76.7%	23.3%	
	Total	64.3%	35.7%	
Anti-dsDNA	positive	45.5%	54.5%	<0.001^c^
	negative	84.8%	15.2%	
	Total	63.4%	36.6%	
Complement consumption	absent	65.7%	34.3%	0.832^c^
	present	63.6%	36.4%	
	Total	64.3%	35.7%	
Hypertension	no	46.2%	53.8%	0.028^c^
	yes	69.8%	30.2%	
	Total	64.3%	35.7%	
Final eGFR (CKD-EPI)	≥ 15mL/min	59.2%	40.8%	0.003^c^
	<15mL/min	100.0%	-	
	Total	64.3%	35.7%	

Notes

^b^Mann-Whitney

^c^Pearson Chi-squared.

Among the patients with class V LN, initial serum creatinine was lower (p = 0.002), initial eGFR was higher (p = 0.003), initial (p < 0.001) and follow-up levels of hematuria (p < 0.001) were lower, and there was less frequent anti-dsDNA positivity (p < 0.001); final eGFR > 15 mL/min was more frequent (p = 0.003).

During the follow-up, 15 (13.4%) patients presented renal function impairment (doubling of serum creatinine). The profile of patients who had worsening of renal function until the last year of follow-up did not present a statistically significant difference in univariate statistical analyses when compared with those who did not.

Thirteen patients (11.6%) progressed to ESRD. In univariate analysis, patients who developed ESRD had higher baseline serum creatinine (p < 0.001) and serum creatinine higher than 1.2 mg/dL (p = 0.001) at presentation; they were also more frequently submitted to dialysis for acute renal injury in the follow-up (p = 0.033), had more proteinuria >3.5 g/24 h during the course of the disease (p = 0.010), more hematuria above 100,000/mL (p = 0.032), and more classes IV (p = 0.003) and V (p = 0.033).

Fifty-one (45.5%) patients had a score of at least 1 for renal damage in SDI. Of those 51 patients who had kidney damage, 29 (56.8%) scored for having eGFR <50 mL/min, 19 (37.2%) scored for maintaining proteinuria ≥ 3.5 g/24 h, and 13 (25.4%) scored for ESRD. Thirty-four patients were followed-up for a longer period and had information for evaluation of SDI renal damage in 5 and 10 years, and 4 (11.8%) had an increase in the score over time. In the inferential comparison of the SDI scores between 5 and 10 years, a statistically significant increase was found in such scores (p = 0.046).

The final SDI renal damage score was also correlated with the profile of the patients in order to identify associated risk factors (Table 3). Univariate inferential results revealed that patients with baseline serum creatinine >1.2 mg/dL (p < 0.001), with baseline eGFR <75 mL/min (p < 0.001), who were submitted to dialysis for acute renal injury (p = 0.002), with proteinuria >3.5 g/24 h during the course of the disease (p = 0.013), with class IV LN (p = 0.010), who used intravenous cyclophosphamide (p = 0.010), and who did not use antimalarials (p = 0.003) had the highest SDI renal damage scores. The final SDI score correlated with initial serum creatinine levels (Spearman correlation coefficient, s = 0.406, p < 0.001). Multivariate analysis demonstrated that the factors that were independently correlated with the final SDI were eGFR<75 mL/min at presentation (p < 0.001) and proteinuria >3.5 g/24 h during the course of the disease (p < 0.001).

**Table 3:** Distribution of the demographic, clinical, and laboratory data of the patients with lupus nephritis according to the final sdi renal damage score

		SDI renal damage score				
		0	I	II	III	p
Gender	male	66.7%	5.6%	16.7%	11.1%	0.518^b^
	female	52.1%	28.7%	7.4%	11.7%	
	Total	54.5%	25.0%	8.9%	11.6%	
Race	white	54.4%	24.6%	7.0%	14.0%	0.894^b^
	non-white	55.3%	23.4%	10.6%	10.6%	
	Total	54.8%	24.0%	8.7%	12.5%	
Hypertension	no	65.4%	19.2%	-	15.4%	0.285^b^
	yes	51.2%	26.7%	11.6%	10.5%	
	Total	54.5%	25.0%	8.9%	11.6%	
Anti-dsDNA	positive	58.2%	27.3%	7.3%	7.3%	0.235^b^
	negative	50.0%	23.9%	8.7%	17.4%	
	Total	54.5%	25.7%	7.9%	11.9%	
Complement consumption	absent	48.6%	25.7%	11.4%	14.3%	0.342^b^
	present	57.1%	24.7%	7.8%	10.4%	
	Total	54.5%	25.0%	8.9%	11.6%	
Initial serum creatinine	≤ 1.2 mg/dL	67.7%	24.2%	4.8%	3.2%	<0.001^b^
	> 1.2 mg/dL	38.0%	26.0%	14.0%	22.0%	
	Total	54.5%	25.0%	8.9%	11.6%	
Initial eGFR	< 75 mL/min	38.7%	27.4%	14.5%	19.4%	<0.001^b^
	≥ 75 mL/min	74.0%	22.0%	2.0%	2.0%	
	Total	54.5%	25.0%	8.9%	11.6%	
Dialysis due to ARF	no	60.6%	22.3%	8.5%	8.5%	0.002^b^
	yes	22.2%	38.9%	11.1%	27.8%	
	Total	54.5%	25.0%	8.9%	11.6%	
Initial 24-h proteinuria	0.2 - 2 g/24h	54.3%	17.1%	8.6%	20.0%	0.798^d^
	2 - 3.5g/24h	45.5%	50.0%	–	4.5%	
	> 3.5g/24h	58.2%	20.0%	12.7%	9.1%	
	Total	54.5%	25.0%	8.9%	11.6%	
24h proteinuria during follow-up	≤ 3.5 g/24h	67.7%	32.3%	–	–	0.013^b^
	> 3.5 g/24h	49.4%	22.2%	12.3%	16.0%	
	Total	54.5%	25.0%	8.9%	11.6%	
Initial hematuria (if present)	10,000 - 100,000/mL	60.9%	23.9%	6.5%	8.7%	0.148^b^
	>100,000/mL	48.1%	25.0%	11.5%	15.4%	
	Total	54.1%	24.5%	9.2%	12.2%	
Hematuria during follow-up	≤ 100,000	56.4%	30.8%	10.3%	2.6%	0.375^b^
	>100,000	53.4%	21.9%	8.2%	16.4%	
	Total	54.5%	25.0%	8.9%	11.6%	

Abbreviations - ARF: acute renal failure; eGFR: estimated glomerular filtration rate (CKD-EPI).

Notes

^b^Mann-Whitney

^d^Kruskal-Wallis.

## Discussion

The development of CKD and ESRD in LN is an important concern in the follow-up of these patients. In 1997, Medicare data in the United States indicated that LN was the main diagnosis in 2% of dialysis patients and in 5% of renal transplant patients^26^. The risk of CKD in patients with SLE has decreased in the last decades, coinciding with the evolution of treatment, a greater knowledge of the disease, and earlier diagnosis. From 1970 to the mid-1990s, the absolute risk of CKD fell by about 10%^27^. There was also an improvement in the incidence of ESRD in patients with LN over time, from approximately 16% in the 1970s and 1980s to 11% in the 1990s and 2000s, but there are indications that this incidence has since reached a plateau^24^.

Some previous studies have focused on finding risk factors related to renal function impairment and ESRD, and the results tend to vary considerably^12,13,14,15,16,27,29,30,31^. Our study evaluated demographic characteristics, clinicopathological correlations, and risk factors associated with renal outcomes in a cohort of Brazilian patients with LN, who were followed for a median time of 8.3 years. The mean initial serum creatinine of our population was 1.62 mg/dL, which shows that a considerable number of our patients started follow-up with impaired renal function. Previous studies in patients with SLE considered that serum creatinine was altered at baseline when its value was equal to or greater than 1.2 mg/dL, and this occurred in 32 to 39.3% of the cases on average ^27,28^. In our sample, 50% of patients had an initial serum creatinine ≥1.2 mg/dL. Similarly, almost half of the patients (49.1%) had proteinuria >3.5 g/24 h at onset and the vast majority of the patients (72.3%) had proteinuria >3.5 g/24 h at least once during follow-up. Undoubtedly, all these aspects demonstrate the severity of the presentation of LN in the population that we evaluated. It is important to remember that the Division of Nephrology (Section of Glomerulopathies) of the UNIFESP is part of a large tertiary hospital and receives patients from all over the country, often referred due to serious and complex conditions, which may partly explain the type of presentation reported, in addition to the greater severity of the disease.

Nevertheless, we found a relatively low frequency of progressive CKD in our population, consistent with previous studies. Up to the last year of follow-up, baseline serum creatinine doubled in 13.4% of patients. In addition, the profile of patients who had worsening renal function did not present a significant difference when compared to those who did not worsen. Previous studies report a widely variable CKD incidence of 8.9 to 44%^8,13,25,27,29,32,33^.

When analyzing the SDI renal damage score, 45.5% of the patients had a score of at least 1. This index was much higher than that found when studying a population with SLE in general and not specifically with LN as we did. In previous studies with SLE, renal involvement occurred in 11 to 23.3% of the patients^24,34^. Our data shows that a baseline serum creatinine > 1.2 mg/dL, baseline eGFR <75 mL/min, dialysis due to acute renal injury during follow-up, proteinuria >3.5 g/24 h during the course of the disease, class IV LN, use of intravenous cyclophosphamide, and non-use of antimalarials were risk factors for renal damage in SDI in the univariate analysis. However, multivariate analysis demonstrated that only eGFR <75 mL/min at the onset and proteinuria >3.5 g/24h along the course of the disease remained as independent risk factors. The final SDI score correlated with initial serum creatinine levels.

There is already sufficient evidence that the damage measured by SDI is a predictor of survival as well as of future damage^25,35,36^ and that the domain of renal damage has a greater relationship with decrease in survival than other domains^25,36^. Both the initial renal function deficit and proteinuria have been pointed out countless times as predictors of worse renal outcome, as confirmed in the present study. Studies with large cohorts^31,32^ have concluded that reduction of proteinuria is the factor that exerts the greatest influence on renal prognosis. Minor trials identified nephrotic levels of proteinuria during the course of disease as an independent factor to define renal prognosis^15,37^. The vast majority of studies have shown that the initial serum creatinine or the presence of initial renal function deficit are risk factors for progression to ESRD^12,13,16,29,30^. Thus, there is a consensus that proliferative classes deserve a more aggressive treatment when there is elevated serum creatinine and/or nephrotic proteinuria^13^.

Other risk factors considered to be predictors of renal survival, such as gender and histological classes of LN, were not associated with worsening renal function in our study. In fact, even LN class IV did not remain a predictor of worse prognosis in the multivariate analysis in our sample. Although proliferative classes are often cited as a risk factor, there are some important trials that have not found such association^8,27^. It is possible that in recent years, the early identification and institution of intensive immunosuppressive treatment adopted in such cases have changed the role of this variable in the current course of LN. It is of note that the use of antimalarials has also gained support recently, with evidence suggesting association of a lower rate of kidney damage and a lower rate of ESRD^18,19^. Nevertheless, we found this association only in the univariate analysis.

The development of ESRD, defined as eGFR <15 mL/min, independent of the need of dialysis or renal transplantation, occurred in 11.6% of the patients. Data on the incidence of ESRD vary considerably, as well as the characteristics of the populations studied, making it difficult to compare populations. In general, reports about the development of ESRD in LN patients range from 8 to 30%^12,25,27,28,38,39,40^. Patients in our sample that progressed to ESRD presented significantly higher initial serum creatinine, as well as more frequent serum creatinine levels >1.2 mg/dL, proteinuria >3.5 g/24 h during the course of the disease, a greater need of dialysis for acute renal injury during follow-up, hematuria >100,000/mL during the course of the disease, more cases of class IV and V LN, and less frequent use of azathioprine and antimalarials.

Change in early renal function has been well established as a predictor of the development of ESRD^12,29,30^. Other studies also related proteinuria to nephrotic levels throughout the follow-up with the development of ESRD^37,41,42^. It is not uncommon for LN class IV to be considered the worst prognostic factor for renal survival^15,16,29^, but pure class V LN is not cited as a prognostic factor for the development of ESRD, as we observed. Many studies of renal survival have pointed to the nephritic flare and lack of remission with treatment during follow-up as predictors for development of ESRD^13,29^. In our study, we did not evaluate the criteria for nephritic flare as a whole or any remission criteria due to the difficulty of defining such criteria with only the available retrospective data. However, it is possible that detection of hematuria levels >100,000/mL during follow-up as a predictor of renal prognosis could correspond to the nephritic flares as prognostic factors in such cases.

Among the limitations of our study is the fact that it is a retrospective study. The different treatments carried out were also not separated according to the induction and maintenance phases, and comparisons between the most common classes were not included, particularly between proliferative (III and IV) and pure membranous classes.

In conclusion, the population of the present study was characterized by an expressive renal impairment at LN presentation. Nevertheless, worsening of renal function (defined as the doubling of serum creatinine) had a relatively low frequency compared to other studies, possibly reflecting early diagnosis and treatment of the predominant severe classes of LN. The main determinants of renal outcome were baseline eGFR <75 mL/min and proteinuria >3.5 g/24h during follow-up. Cumulative renal damage measured by SDI in the present study was associated with such important markers of renal involvement, demonstrating that it might be a useful tool to assess chronic injury and the need for preventing disease complications. Thus, an earlier diagnosis and better management of LN, especially in cases with high levels of proteinuria, may help to improve the prognosis of such disease.
