# Supplementary material for: Renal outcomes in the long-term follow-up of lupus nephritis
Source: J Bras Nefrol. 2025 Apr 14;47(3):e20240118. doi: 10.1590/2175-8239-JBN-2024-0118en (PMC12004670; doi:10.1590/2175-8239-JBN-2024-0118en)
Supplement: Translation

# Translation: Desfechos renais no acompanhamento de longo prazo da nefrite lúpica

**DOI:** 10.1590/2175-8239-JBN-2024-0118pt

**Authors:** Sabrina Bonvino Polycarpo, Gianna Mastroianni Kirsztajn

**Affiliations:** 1 Universidade Federal de São Paulo, Departamento de Medicina (Nefrologia), São Paulo, SP, Brasil.

**Keywords:** Nefrite Lúpica;, Lúpus Eritema-toso Sistêmico, Insuficiência Renal Crônica, Falência Renal Crônica, Antimaláricos, Creatinina, Taxa de Filtração Glomerular

## Abstract

**Introdução:**

Nefrite lúpica (NL) é uma das manifestações mais comuns e graves do lúpus eritematoso sistêmico (LES), reconhecida como o maior preditor de mau prognóstico. O objetivo deste estudo é analisar características demográficas, correlações clinicopatológicas e fatores de risco associados a desfechos renais no acompanhamento de longo prazo de pacientes com NL.

**Método:**

Estudo de coorte observacional retrospectivo de 112 pacientes com NL comprovada por biópsia, acompanhados de julho/1985 a agosto/2015. Avaliamos fatores de risco associados a comprometimento renal (dobrar o valor da creatinina sérica), doença renal em estágio terminal (DRET, TFGe <15 mL/min) e dano renal cumulativo utilizando o Índice de Danos (ID) da Systemic Lupus International Collaborating Clinics/ACR.

**Resultados:**

O tempo mediano de acompanhamento foi 8,3 anos. Houve predominância de NL classe IV (64,3%), isoladamente ou em combinação e também um aumento estatisticamente significativo no escore renal do ID entre 5 e 10 anos de acompanhamento. Na análise multivariada, o ID final correlacionou-se com TFGe inicial <75 mL/min (p < 0,001) e proteinúria >3,5 g/24 h ao longo da doença (p < 0,001), superando parâmetros identificados na análise univariada como uso de ciclofosfamida (p = 0,010) e não uso de antimaláricos (p = 0,003); 13,4% dos pacientes evoluíram para comprometimento da função renal e 11,6% para DRET.

**Discussão:**

O dano renal acumulado, avaliado pelo ID, foi um achado frequente. Os principais determinantes do desfecho renal foram TFGe basal <75 mL/min e proteinúria >3,5 g/24 h durante o acompanhamento. Assim, diagnóstico precoce e melhor manejo da NL, especialmente em caso de altos níveis de proteinúria, podem contribuir para melhora do prognóstico.

## Introdução

O lúpus eritematoso sistêmico (LES) é uma doença autoimune crônica e multissistêmica do tecido conjuntivo^1^. O envolvimento renal no LES, a nefrite lúpica (NL), é frequente e constitui também uma das manifestações mais graves de LES. Afeta até 60% dos adultos com a doença; a incidência depende da população estudada, sendo maior em asiáticos, afrodescendentes e hispânicos^2−4^. A NL é reconhecida como o principal preditor de mau prognóstico, uma vez que está associada a um aumento significativo da mortalidade^2,4−6^. Há cerca de 50 anos, a sobrevida mediana em cinco anos de pacientes com NL era de 44%; melhorou drasticamente para aproximada-mente 82% na década de 1990. Essa redução na mortalidade deve-se ao melhor conhecimento da doença, o que leva a um diagnóstico mais precoce, e a um manejo terapêutico mais eficaz e agressivo^2,4,7^.

Apesar de melhorar o curso da doença, o tratamento pode ser prolongado, potencialmente tóxico e difícil de planejar e manter^8,9^. Tanto o tratamento quanto a atividade da doença ao longo do tempo podem causar danos permanentes a diferentes órgãos, levando à disfunção, como a doença renal crônica (DRC) no caso da NL. Para alguns autores^9,10^, a identificação de preditores de danos cumulativos é de grande importância para melhorar os desfechos do LES. Nesse contexto, o Índice de Danos (ID) da *Systemic Lupus International Collaborating Clinics/ACR* é um instrumento validado para medir o dano irreversível em pacientes com LES^9^. De fato, a sobrevida em curto prazo de pacientes com LES melhorou, mas danos irreversíveis aos órgãos se acumulam durante o curso da doença, contribuindo para o comprometimento da qualidade de vida e o aumento da mortalidade em longo prazo. Assim, o manejo do paciente deve ter como objetivo não apenas aumentar a sobrevida, mas também reduzir a morbidade causada pela doença e sua terapia^10^. Isso pode ser, em parte, avaliado por índices como o ID, que podem fornecer informações adicionais no acompanhamento de pacientes com NL.

A incidência média de doença renal em estágio terminal (DRET) em 10 anos de acompanhamento é de 15% e pode chegar a 25−30% em 15 anos^11,12,13,14,15^. A ausência de remissão da NL com o tratamento é provavelmente um dos mais importantes preditores de progressão para DRC. Outros fatores de risco para o comprometimento renal também foram estabelecidos e incluem sexo masculino, baixo nível socioeconômico, etnia (ser afrodescendente ou hispânico), presença de crescentes em mais de 50% dos glomérulos na biópsia renal, classes proliferativas (III e IV) e tratamento com corti-coides isoladadamente, não utilização de antimaláricos, creatinina sérica inicial elevada, proteinúria nefrótica, hipertensão arterial sistêmica, anemia e consumo de complemento^16,17,18,19^. Tais características tendem a variar de acordo com cada estudo, uma vez que há diferenças nas definições dos desfechos, nas populações e na duração do acompanhamento, dificultando a extrapolação dos achados.

Estudos anteriores sobre desfechos em pacientes brasileiros com NL demonstraram que a proteinúria inicial e os níveis de creatinina sérica, assim como a resposta inicial ao tratamento e a cronicidade na biópsia renal, estiveram relacionados a piores desfechos^20,21,22^. Analisamos as características demográficas, as correlações clinicopatológicas e os fatores de risco associados aos desfechos renais em um acompanhamento de longo prazo, incluindo perda da função renal, DRET e danos cumulativos em uma coorte longitudinal de pacientes com NL comprovada por biópsia.

## Métodos

Foram analisados dados retrospectivos de prontuários médicos de pacientes com diagnóstico de LES - de acordo com os critérios revisados do *American College of Rheumatology* (ACR) - e diagnóstico histopatológico de NL, acompanhados na Disciplina de Nefrologia (Seção de Glomerulopatias) da Universidade Federal de São Paulo (UNIFESP), um centro de referência terciário e acadêmico em São Paulo, Brasil. Os pacientes incluídos foram acompanhados de julho de 1985 a agosto de 2015. A NL foi classificada de acordo com as classificações da Organização Mundial da Saúde (OMS) e da *International Society of Nephrology/Renal Pathology Society* (ISN/RPS)^23^. Foram incluídos pacientes de ambos os sexos, com idade igual ou superior a 12 anos e com um tempo de acompanhamento igual ou superior a um ano.

As seguintes variáveis foram avaliadas: idade no início do acompanhamento, sexo, pressão arterial, creatinina sérica, proteinúria, hematúria, anticorpos antinucleares e anti-DNA de dupla hélice, consumo de complemento, manifestações laboratoriais e clínicas do LES, taxa de filtração glomerular estimada (TFGe) determinada por meio da fórmula *Chronic Kidney Disease Epidemiology Collaboration* (CKD-EPI)^24^ e laudos de biópsia. O tipo e a duração do tratamento, bem como o período de início das infecções, também foram registrados.

O comprometimento renal foi definido como alcançar pelo menos a duplicação do valor da creatinina sérica e a DRET como TFGe <15 mL/min (DRC estágio 5) ou necessidade de terapia renal substitutiva. Também avaliamos o dano renal utilizando o ID^9^. Esse instrumento já foi validado e aplicado em diversas populações e etnias. Ele avalia os danos não reversíveis, relacionados ao LES e seu tratamento, em 12 órgãos ou sistemas^9,10,25^. O dano no sistema renal, de acordo com o ID, foi definido como um dos seguintes: 1) TFG estimada <50 mL/min (escore: 1); 2) proteinúria persistente na faixa nefrótica ≥ 3,5 g/24 h (escore: 1); e DRET (escore: 3). Todos os pacientes tiveram o ID avaliado em 1 ano e ao final do acompanhamento; 34 pacientes que foram acompanhados por um período mais longo também tiveram o ID avaliado em 5 e 10 anos.

As análises estatísticas foram inicialmente realizadas de forma descritiva (médias, medianas, porcentagens). Em seguida, testes inferenciais foram aplicados quando necessário e citados na seção Resultados. Valores de p inferiores a 0,05 foram considerados estatisticamente significativos. As análises foram realizadas com o software R, versão 3.0.2. (*R Core Team*, 2016).

## Resultados

Foram incluídos 112 pacientes, com um tempo mediano de acompanhamento de 99,9 meses (intervalo de 12 a 322). A idade média foi de 31 ± 10,8 anos, 83,9% eram do sexo feminino e 54,8% eram brancos. As características basais são apresentadas na Tabela 1.

**Tabela 1:** Características demográficas e clínicas dos pacientes com nefrite lúpica (n = 112)

Idade média ± dp (anos)	31 ± 10,8
Sexo (%)	
Masculino	16,1
Feminino	83,9
Tempo médio de acompanhamento (meses)	99,9 (12-322)
Raça (%)	
Branca	54,8
Não branca	45,2
Creatinina sérica média basal ± DP (mg/dL)	1,62 ± 1,30
TFGe média ± DP (mL/min/1,73 m^2^)	71,1 ± 40,1
TFGe final (%)	
≥ 15 mL/min	87,5
< 15 mL/min	12,5
Proteinúria média ± DP (g/24 h)	4,15 ± 3,34
Proteinúria durante acompanhamento (%)	
> 3,5 g/24 h	72,3
≤ 3,5 g/24 h	27,7
Hematúria na apresentação (%)	87,5
Hematúria durante acompanhamento (%)	
≤ 100.000/mL	34,8
> 100.000/mL	65,2
Diálise por IRA durante acompanhamento (%)	16,1
Hipertensão arterial (%)	76,8
Anemia (%)	74,1
Trombocitopenia (%)	8,0
Leucopenia (%)	15,2
Consumo de complemento (%)	68,8
Anti-DNA (%)	15,2

As manifestações clínicas predominantes do LES associadas à NL foram as hematológicas (80,4%), com destaque para anemia (74,1%), seguidas por consumo de complemento (68,8%) e acometimento articular (67%). A hematúria >100.000/mm^3^ na apresentação da doença renal ocorreu em 52 pacientes (53,1% dos pacientes com hematúria) e, ao final do acompanhamento, em seis pacientes (11,1% daqueles com hematúria nesse estágio). Na apresentação, o perfil laboratorial revelou creatinina sérica elevada (média de 1,62 mg/dL) e proteinúria >3,5 g/24 h em 49% dos pacientes e, em algum momento do curso da doença, em 72% dos pacientes.

A distribuição das classes histológicas da NL ocorreu da seguinte forma: 3,57% classe II, 7,14% classe III, 64,29% classe IV, 23,21% classe V e 1,79% classe VI. Uma combinação de classes ocorreu em 11 pacientes, todos com classe IV, e 8 deles eram classe IV+V. Para simplificar a análise, esses últimos foram todos categorizados como classe IV.

A hipertensão arterial foi diagnosticada em 76,8% dos pacientes, exigindo apenas um ou dois medicamentos anti-hipertensivos para controle na maioria dos casos. Todos os pacientes haviam recebido corticosteroides durante o acompanhamento; 53,6% dos pacientes receberam ciclofosfamida intravenosa, 19,6% ciclofosfamida oral, 47,3% azatioprina, 41,1% micofenolato (mofetil ou sódico) e 62,5% antimaláricos (cloroquina ou hidroxicloroquina). O tratamento com azatioprina foi mais comum em pacientes com NL classe II (p = 0,047). Na classe IV, os tratamentos mais utilizados foram a ciclofosfamida intravenosa (p < 0,001) e o micofenolato (p = 0,003). Esses tratamentos foram menos utilizados entre os pacientes da classe V (p = 0,006 e p = 0,011, respectivamente). Infecções do trato respiratório superior foram associadas ao uso de ciclofosfamida oral (p = 0,034).

Em comparação com as demais classes de NL, a classe IV (Tabela 2) esteve associada a uma pior função renal no início do estudo, maior creatinina sérica inicial (p < 0,001) e menor TFGe inicial (p < 0,001), bem como à detecção mais frequente de critérios de atividade renal, como hematúria >100.000/mL inicial e durante o acompanhamento (p < 0,001), atividade imunológica com maior frequência de positividade para anti-DNA (p < 0,001) e detecção mais frequente de hipertensão arterial (p = 0,028). A classe IV também apresentou maior incidência de DRET no final do acompanhamento quando comparada com as outras classes (p = 0,003).

**Tabela 2:** Distribuição do perfil demográfico, clínico e laboratorial dos pacientes com nefrite lúpica classe iv

		NL classe IV		
		sim	não	P
Sexo	masculino	61,1%	38,9%	0,759^c^
	feminino	64,9%	35,1%	
	Total	64,3%	35,7%	
Idade (anos)	n	72	40	0,417^b^
	média	30,3	32,3	
	mediana	28,0	30,5	
	mínimo	13,0	14,0	
	máximo	54,0	73,0	
	desvio padrão	10,3	11,6	
Raça	branca	71,9%	28,1%	0,078^c^
	não-branca	55,3%	44,7%	
	Total	64,4%	35,6%	
Creatinina sérica inicial (mg/dL)	n	72	40	<0,001^b^
	média	2,03	0,88	
	mediana	1,40	0,80	
	mínimo	0,43	0,44	
	máximo	7,00	1,84	
	desvio padrão	1,45	0,33	
TFGe inicial (mL/min)	n	72	40	<0,001^b^
	média	56,34	9777	
	mediana	50,55	99,25	
	mínimo	7,30	42,70	
	máximo	145,40	151,00	
	desvio padrão	37,12	30,54	
Proteinúria de 24h durante acompanhamento	≤ 3,5g/24h	58,1%	41,9%	0,395^c^
	>3,5g/24h	66,7%	33,3%	
	Total	64,3%	35,7%	
Proteinúria de 24h inicial	0,2 a 2g/24h	62,9%	37,1%	0,219^c^
	2 a 3,5g/24h	50,0%	50,0%	
	>3,5g/24h	70,9%	29,1%	
	Total	64,3%	35,7%	
Hematúria inicial (se presente)	10.000 a 100.000/mL	50,0%	50,0%	<0,001^c^
	>100.000/mL	84,6%	15,4%	
	Total	68,4%	31,6%	
Hematúria durante acompanhamento	≤ 100.000/mL	41,0%	59,0%	<0,001^c^
	>100.000/mL	76,7%	23,3%	
	Total	64,3%	35,7%	
Anti-DNA	positivo	45,5%	54,5%	<0,001^c^
	negativo	84,8%	15,2%	
	Total	63,4%	366%	
Consumo de complemento	ausente	65,7%	34,3%	0,832^c^
	presente	63,6%	36,4%	
	Total	64,3%	35,7%	
Hipertensão arterial	não	46,2%	53,8%	0,028^c^
	sim	69,8%	30,2%	
	Total	64,3%	35,7%	
TFGe final (CKD-EPI)	≥ 15mL/min	59,2%	40,8%	0,003^c^
	<15mL/min	100,0%	-	
	Total	64,3%	35,7%	

Notas

^b^Mann-Whitney

^c^Qui-quadrado de Pearson.

Entre os pacientes com NL classe V, a creatinina sérica inicial foi menor (p = 0,002), a TFGe inicial foi maior (p = 0,003), os níveis iniciais (p < 0,001) e de acompanhamento de hematúria (p < 0,001) foram menores e houve positividade para anti-DNA menos frequentemente (p < 0,001); a TFGe final > 15 mL/min foi mais frequente (p = 0,003).

Durante o acompanhamento, 15 (13,4%) pacientes apresentaram comprometimento da função renal (duplicação da creatinina sérica). O perfil dos pacientes que apresentaram piora da função renal até o último ano de acompanhamento não demonstrou diferença estatisticamente significativa nas análises estatísticas univariadas quando comparado aos que não apresentaram piora.

Treze pacientes (11,6%) evoluíram para DRET. Na análise univariada, os pacientes que desenvolveram DRET apresentaram creatinina sérica basal mais elevada (p < 0,001) e creatinina sérica superior a 1,2 mg/dL (p = 0,001) na apresentação; também foram mais frequentemente submetidos à diálise por injúria renal aguda no acompanhamento (p = 0,033), apresentaram mais proteinúria >3,5 g/24 h durante o curso da doença (p = 0,010), mais hematúria acima de 100.000/mL (p = 0,032) e eram mais frequentemente das classes IV (p = 0,003) e V (p = 0,033).

Cinquenta e um (45,5%) pacientes apresentaram um escore de pelo menos 1 para dano renal no ID. Desses 51 pacientes que apresentaram lesão renal, 29 (56,8%) pontuaram por apresentarem TFGe <50 mL/min, 19 (37,2%) por manterem proteinúria ≥ 3,5 g/24h e 13 (25,4%) por DRET. Trinta e quatro pacientes foram acompanhados por um período mais longo e gerando informações para avaliação do dano renal por meio do ID em 5 e 10 anos, e 4 (11,8%) apresentaram um aumento no escore ao longo do tempo. Na comparação inferencial dos escores do ID entre 5 e 10 anos, foi encontrado um aumento estatisticamente significativo nesses escores (p = 0,046).

O escore final de dano renal do ID também foi correlacionado com o perfil dos pacientes para identificar fatores de risco associados (Tabela 3). Os resultados inferenciais univariados revelaram que pacientes com creatinina sérica basal >1,2 mg/dL (p < 0,001), com TFGe basal <75 mL/min (p < 0,001), submetidos à diálise por injúria renal aguda (p = 0,002), com proteinúria >3.5 g/24 h durante o curso da doença (p = 0,013), com NL classe IV (p = 0,010), que fizeram uso de ciclofosfamida intravenosa (p = 0,010) e que não utilizaram antimaláricos (p = 0,003) apresentaram os escores mais elevados de dano renal pelo ID. O escore final do ID correlacionou-se com os níveis iniciais de creatinina sérica (coeficiente de correlação de Spearman, s = 0,406; p < 0,001). A análise multivariada demonstrou que os fatores que foram independentemente correlacionados com o ID final foram TFGe<75 mL/min na apresentação (p < 0,001) e proteinúria >3,5 g/24 h durante o curso da doença (p < 0,001).

**Tabela 3:** Distribuição dos dados demográficos, clínicos e laboratoriais dos pacientes com nefrite lúpica de acordo com o escore final de dano renal do id

		Escore de dano renal do ID				
		0	I	II	III	p
Sexo	masculino	66,7%	5,6%	16,7%	11,1%	0,518^b^
	feminino	52,1%	28,7%	7,4%	11,7%	
	Total	54,5%	25,0%	8,9%	11,6%	
Raça	branca	54,4%	24,6%	7,0%	14,0%	0,894^b^
	não-branca	55,3%	23,4%	10,6%	10,6%	
	Total	54,8%	24,0%	8,7%	12,5%	
Hipertensão arterial	não	65,4%	19,2%	-	15,4%	0,285^b^
	sim	51,2%	26,7%	11,6%	10,5%	
	Total	54,5%	25,0%	8,9%	11,6%	
Anti-DNA	positivo	58,2%	27,3%	7,3%	7,3%	0,235^b^
	negativo	50,0%	23,9%	8,7%	17,4%	
	Total	54,5%	25,7%	7,9%	11,9%	
Consumo de complemento	ausente	48,6%	25,7%	11,4%	14,3%	0,342^b^
	presente	57,1%	24,7%	7,8%	10,4%
	Total	54,5%	25,0%	8,9%	11,6%
Creatinina sérica inicial	≤ 1,2 mg/dL	67,7%	24,2%	4,8%	3,2%	<0,001^b^
	> 1,2 mg/dL	38,0%	26,0%	14,0%	22,0%	
	Total	54,5%	25,0%	8,9%	11,6%	
TFGe inicial	< 75 mL/min	38,7%	27,4%	14,5%	19,4%	<0,001^b^
	≥ 75 mL/min	74,0%	22,0%	2,0%	2,0%	
	Total	54,5%	25,0%	8,9%	11,6%	
Diálise devido à IRA	não	60,6%	22,3%	8,5%	8,5%	0,002^b^
	sim	22,2%	38,9%	11,1%	27,8%	
	Total	54,5%	25,0%	8,9%	11,6%	
Proteinúria de 24h inicial	0,2 a 2 g/24h	54,3%	17,1%	8,6%	20,0%	0,798^d^
	2 a 3,5g/24h	45,5%	50,0%	–	4,5%	
	> 3,5g/24h	58,2%	20,0%	12,7%	9,1%	
Total	54,5%	25,0%	8,9%	11,6%
Proteinúria de 24h durante acompanhamento	≤ 3,5 g/24h	67,7%	32,3%	–	–	0,013^b^
	> 3,5 g/24h	49,4%	22,2%	12,3%	16,0%	
	Total	54,5%	25,0%	8,9%	11,6%	
Hematúria inicial (se presente)	10.000 a 100.000/mL	60,9%	23,9%	6,5%	8,7%	0,148^b^
	>100.000/mL	48,1%	25,0%	11,5%	15,4%	
	Total	54,1%	24,5%	9,2%	12,2%	
Hematúria durante acompanhamento	≤ 100.000	56,4%	30,8%	10,3%	2,6%	0,375^b^
	>100.000	53,4%	21,9%	8,2%	16,4%	
	Total	54,5%	25,0%	8,9%	11,6%	

Abreviações - IRA: insuficiência renal aguda; TFGe: taxa de filtração glomerular estimada (CKD-EPI).

Notas

^b^Mann-Whitney

^d^Kruskal-Wallis.

## Discussão

O desenvolvimento de DRC e DRET na NL é uma preocupação relevante no acompanhamento desses pacientes. Em 1997, dados do *Medicare* nos Estados Unidos indicaram que a NL foi o principal diagnóstico em 2% dos pacientes em diálise e em 5% dos pacientes submetidos a transplante renal^26^. O risco de DRC em pacientes com LES tem diminuído nas últimas décadas, coincidindo com a evolução do tratamento, um maior conhecimento da doença e o diagnóstico mais precoce. De 1970 a meados da década de 1990, o risco absoluto de DRC reduziu em cerca de 10%^27^. Houve também uma queda na incidência de DRET em pacientes com NL ao longo do tempo, de aproximadamente 16% nas décadas de 1970 e 1980 para 11% nas décadas de 1990 e 2000; porém, há indícios de que essa incidência tenha atingido um *plateau* desde então^24^.

Alguns estudos anteriores se concentraram em identificar fatores de risco relacionados ao compro-metimento da função renal e à DRET, e os resultados tendem a variar consideravelmente^12,13,14,15,16,27,29,30,31^. Nosso estudo avaliou as características demográficas, correlações clinicopatológicas e fatores de risco associados a desfechos renais em uma coorte de pacientes brasileiros com NL, que foram acompanhados por um período mediano de 8,3 anos. A creatinina sérica inicial média de nossa população foi de 1,62 mg/dL, o que demonstra que um número considerável de nossos pacientes iniciou o acompanhamento com função renal prejudicada. Estudos anteriores em pacientes com LES consideraram que a creatinina sérica estava alterada no início do estudo quando seu valor era igual ou superior a 1,2 mg/dL, e isso ocorreu em 32 a 39,3% dos casos, em média^27,28^. Em nossa amostra, 50% dos pacientes apresentaram creatinina sérica inicial ≥1,2 mg/dL. Da mesma forma, quase metade dos pacientes (49,1%) apresentou proteinúria >3,5 g/24 h no início e a grande maioria dos pacientes (72,3%) apresentou proteinúria >3,5 g/24 h pelo menos uma vez durante o acompanhamento. Sem dúvida, todos esses aspectos demonstram a gravidade da apresentação da NL na população que avaliamos. É importante lembrar que a Disciplina de Nefrologia (Seção de Glomerulopatias) da UNIFESP faz parte de um grande hospital terciário e recebe pacientes de todo o país, muitas vezes encaminhados devido a quadros graves e complexos, o que pode explicar em parte o tipo de apresentação relatado, além da maior gravidade da doença.

No entanto, encontramos uma frequência relativamente baixa de DRC progressiva em nossa população, consistente com estudos anteriores. Até o último ano de acompanhamento, a creatinina sérica basal duplicou em 13,4% dos pacientes. Além disso, o perfil dos pacientes que apresentaram piora da função renal não demonstrou diferença significativa em relação aos que não apresentaram piora. Estudos anteriores relatam uma incidência de DRC amplamente variável de 8,9 a 44%^8,13,25,27,29,32,33^.

Ao analisar o escore de dano renal do ID, 45,5% dos pacientes apresentaram um escore de pelo menos 1. Esse índice foi muito maior do que o encontrado ao estudar uma população com LES de modo geral, e não especificamente com NL, como fizemos. Em estudos prévios com LES, o envolvimento renal ocorreu em 11 a 23,3% dos pacientes^24,34^. Nossos dados mostram que creatinina sérica basal > 1,2 mg/dL, TFGe basal <75 mL/min, diálise devido à injúria renal aguda durante o acompanhamento, proteinúria >3,5 g/24 h durante o curso da doença, NL classe IV, uso de ciclofosfamida intravenosa e não uso de antimaláricos foram fatores de risco para dano renal no ID na análise univariada. Entretanto, a análise multivariada demonstrou que apenas a TFGe <75 mL/min no início e a proteinúria >3,5 g/24h ao longo do curso da doença permaneceram como fatores de risco independentes. O escore final do ID correlacionou-se com os níveis iniciais de creatinina sérica.

Já existem evidências suficientes de que o dano medido pelo ID é um preditor de sobrevida, bem como de danos futuros^25,35,36^, e que o domínio do dano renal tem maior relação com a diminuição da sobrevida do que outros domínios^25,36^. Tanto o déficit inicial da função renal quanto a proteinúria têm sido apontados inúmeras vezes como preditores de pior desfecho renal, como confirmado no presente estudo. Estudos com grandes coortes^31,32^ concluíram que a redução da proteinúria é o fator que exerce a maior influência no prognóstico renal. Ensaios menores identificaram níveis nefróticos de proteinúria durante o curso da doença como um fator independente para definir o prognóstico renal^15,37^. A grande maioria dos estudos demonstrou que a creatinina sérica inicial ou a presença de déficit inicial da função renal são fatores de risco de progressão para DRET^12,13,16,29,30^. Dessa forma, existe um consenso de que as classes proliferativas merecem um tratamento mais agressivo quando há creatinina sérica elevada e/ou proteinúria nefrótica^13^.

Outros fatores de risco considerados preditores de sobrevida renal, como sexo e classes histológicas de NL, não foram associados à piora da função renal em nosso estudo. De fato, mesmo a NL classe IV não permaneceu como preditor de pior prognóstico na análise multivariada em nossa amostra. Embora as classes proliferativas sejam frequentemente citadas como um fator de risco, existem alguns estudos importantes que não encontraram tal associação^8,27^. É possível que, nos últimos anos, a identificação precoce e a instituição de tratamento imunossupressor intensivo adotadas nesses casos tenham alterado o papel dessa variável no curso atual da NL. É importante ressaltar que o uso de antimaláricos também ganharam apoio mais recentemente, com evidências que sugerem a associação com uma menor taxa de lesão renal e uma taxa reduzida de DRET^18,19^. No entanto, encontramos essa associação somente na análise univariada.

O desenvolvimento da DRET, definida como TFGe <15 mL/min, independentemente da necessidade de diálise ou transplante renal, ocorreu em 11,6% dos pacientes. Os dados sobre a incidência de DRET variam consideravelmente, assim como as características das populações estudadas, o que dificulta a comparação entre essas populações. De modo geral, os relatos sobre o desenvolvimento da DRET em pacientes com NL variam de 8 a 30%^12,25,27,28,38,39,40^. Os pacientes de nossa amostra que evoluíram para DRET apresentaram creatinina sérica inicial significativamente mais elevada, bem como níveis mais frequentes de creatinina sérica >1,2 mg/dL, proteinúria >3,5 g/24 h durante o curso da doença, maior necessidade de diálise por injúria renal aguda durante o acompanhamento, hematúria >100.000/mL durante o curso da doença, mais casos de NL classes IV e V e uso menos frequente de azatioprina e antimaláricos.

A alteração na função renal inicial foi bem estabelecida como um preditor do desenvolvimento de DRET^12,29,30^. Outros estudos também relacionaram proteinúria em níveis nefróticos ao longo do acompanhamento com o desenvolvimento de DRET^37,41,42^. Não é incomum que a NL classe IV seja considerada o pior fator prognóstico para a sobrevida renal^15,16,29^. No entanto, a NL classe V pura não é citada como um fator prognóstico para o desenvolvimento de DRET, tal como observamos. Muitos estudos de sobrevida renal apontaram a crise nefrítica e a ausência de remissão com o tratamento durante o acompanhamento como preditores para o desenvolvimento de DRET^13,29^. Em nosso estudo, não avaliamos os critérios de crise nefrítica como um todo, nem quaisquer critérios de remissão, devido à dificuldade de defini-los apenas com os dados retrospectivos disponíveis. No entanto, é possível que a detecção de níveis de hematúria >100.000/mL durante o acompanhamento como um preditor de prognóstico renal possa corresponder às crises nefríticas como fatores prognósticos nesses casos.

Dentre as limitações do nosso estudo está o fato de que se trata de um estudo retrospectivo. Os diferentes tratamentos realizados também não foram separados de acordo com as fases de indução e manutenção, e não foram incluídas comparações entre as classes mais comuns, particularmente entre as classes proliferativas (III e IV) e a classe membranosa pura.

Em conclusão, a população do presente estudo foi caracterizada por um comprometimento renal expressivo na apresentação da NL. No entanto, a piora da função renal (definida como dobrar o valor da creatinina sérica) apresentou uma frequência relativamente baixa em comparação com outros estudos, possivelmente refletindo o diagnóstico precoce e o tratamento das classes graves predominantes de NL. Os principais determinantes do desfecho renal foram TFGe basal <75 mL/min e proteinúria >3,5 g/24h durante o acompanhamento. O dano renal cumulativo medido pelo ID no presente estudo foi associado a esses importantes marcadores de envolvimento renal, demonstrando que pode ser uma ferramenta útil para avaliar a injúria crônica e a necessidade de prevenir complicações da doença. Assim, um diagnóstico mais precoce e um melhor manejo da NL, especialmente em casos com níveis elevados de proteinúria, podem ajudar a melhorar o prognóstico dessa doença.
